# Herpes Zoster Ophthalmicus Initially Diagnosed As Cluster Headache, Complicated by Delayed Eruption

**DOI:** 10.7759/cureus.56698

**Published:** 2024-03-22

**Authors:** Hidenori Sanayama, Michito Namekawa, Yoshio Sakiyama, Hitoshi Sugawara

**Affiliations:** 1 Department of Comprehensive Medicine 1, Division of General Medicine, Saitama Medical Center, Jichi Medical University, Saitama, JPN; 2 Department of Health and Social Services, Graduate School, Saitama Prefectural University, Saitama, JPN; 3 Department of Comprehensive Medicine 1, Division of Neurology, Saitama Medical Center, Jichi Medical University, Saitama, JPN

**Keywords:** acute herpes zoster, painful trigeminal neuropathy, hutchinson’s sign, hemicrania continua, cluster headache, herpes zoster ophthalmicus

## Abstract

Herpes zoster ophthalmicus (HZO) manifests as a consequence of the reactivation of the Varicella-zoster virus (VZV) and primarily affects the ophthalmic division of the trigeminal nerve. Identification of the vesicular eruption is central to the diagnostic process; however, the delayed manifestation of this cutaneous phenomenon poses a challenge to timely and accurate diagnosis. This report elucidates the case of a 61-year-old Japanese male with painful trigeminal neuropathy attributed to VZV that was initially diagnosed as cluster headache, mainly due to the delayed cutaneous eruption. Contrary to the expected pattern of cluster headache presentations, there was no discernible fluctuation in headache severity. The transient improvement of symptoms following interventions tailored for cluster headache management, including pure oxygen inhalation and subcutaneous sumatriptan injection, inadvertently contributed to a delay in accurate diagnosis. The importance of distinguishing HZO from cluster headache is emphasized, particularly in cases involving elderly patients or those with persistent cephalo-ophthalmalgia without the characteristic fluctuation of symptoms. In cases where clinical suspicion of HZO is raised, cerebrospinal fluid analysis should be performed. This approach is consistent with the overall goal of facilitating a prompt and accurate diagnosis.

## Introduction

Herpes zoster ophthalmicus (HZO), constituting approximately 10-20% of all herpes zoster (HZ) cases, arises from the reactivation of the varicella-zoster virus (VZV) and primarily affects the ophthalmic division of the trigeminal nerve [1]. Typically, acute painful trigeminal neuropathy and cutaneous eruptions manifest concurrently, albeit with instances of pain preceding rashes by several days. Notably, an extended temporal interval of more than a week between these occurrences is feasible, and in some cases, the characteristic rash may never materialize, denoted as "zoster sine herpete." The diagnosis of such cases can be challenging, and in these circumstances, a polymerase chain reaction (PCR) assay for VZV in cerebrospinal fluid (CSF) serves as a valuable diagnostic tool [2].

The ophthalmic division of the trigeminal nerve further anatomically segregates into the nasociliary, frontal, and lacrimal branches. The nasociliary branch encompasses innervation of both the ipsilateral nasal region and the anterior segment of the eyeball, encompassing critical ocular structures, such as the cornea, iris, sclera, and choroid. Therefore, the presence of herpetic eruptions in the lateral aspect of the nose assumes particular significance as a harbinger of ophthalmic complications in HZO, a phenomenon known as "Hutchinson's sign" [1]. In this report, we detailed a case of HZO that was initially diagnosed as a cluster headache due to the delayed onset of characteristic eruptions.

## Case presentation

A 61-year-old Japanese male, bearing a medical history marked by diabetes mellitus, hypertension, and surgical treatment of pearl tumor tympanitis, which caused sensorineural hearing impairment without any accompanying facial sensory disturbances or palsy, experienced the sudden onset of left-sided headache, accompanied by nausea. The unrelenting headache progressively worsened. Brain computed tomography (CT) scans taken at the first hospital where the patient was seen yielded no remarkable findings, and the administration of both loxoprofen and carbamazepine proved ineffectual.

Two days following the onset of symptoms, the patient sought evaluation at an ophthalmological clinic due to pronounced periorbital edema and continuous worsening ophthalmalgia in his left eye. He received a diagnosis of conjunctivitis, and intraocular pressure was noted to be within the normal range. Importantly, corneal herpes was excluded as a contributing factor. Three days later, as his persistent headaches worsened, he could no longer sit still due to the pain. Subsequently, a diagnosis of cluster headache was established at the previous hospital. A treatment composed of inhaling pure oxygen and receiving subcutaneous sumatriptan injection provided temporary relief. However, oral verapamil and amitriptyline failed to produce any beneficial effects.

Two days later, vesicular eruptions emerged on his left forehead and the dorsum of his nose. About three days later, at a follow-up visit at the previous hospital, the rash was accompanied by scabs, and he was diagnosed with shingles. His ability to move the left eye was partially constrained in all directions with constant double vision. An oral prednisolone regimen at a dose of 40 mg per day was initiated, resulting in a rapid improvement in his headache. However, his visual acuity deteriorated, and he was transferred to our hospital for further examination of the cause of his vision loss and for further management.

**Figure 1:**
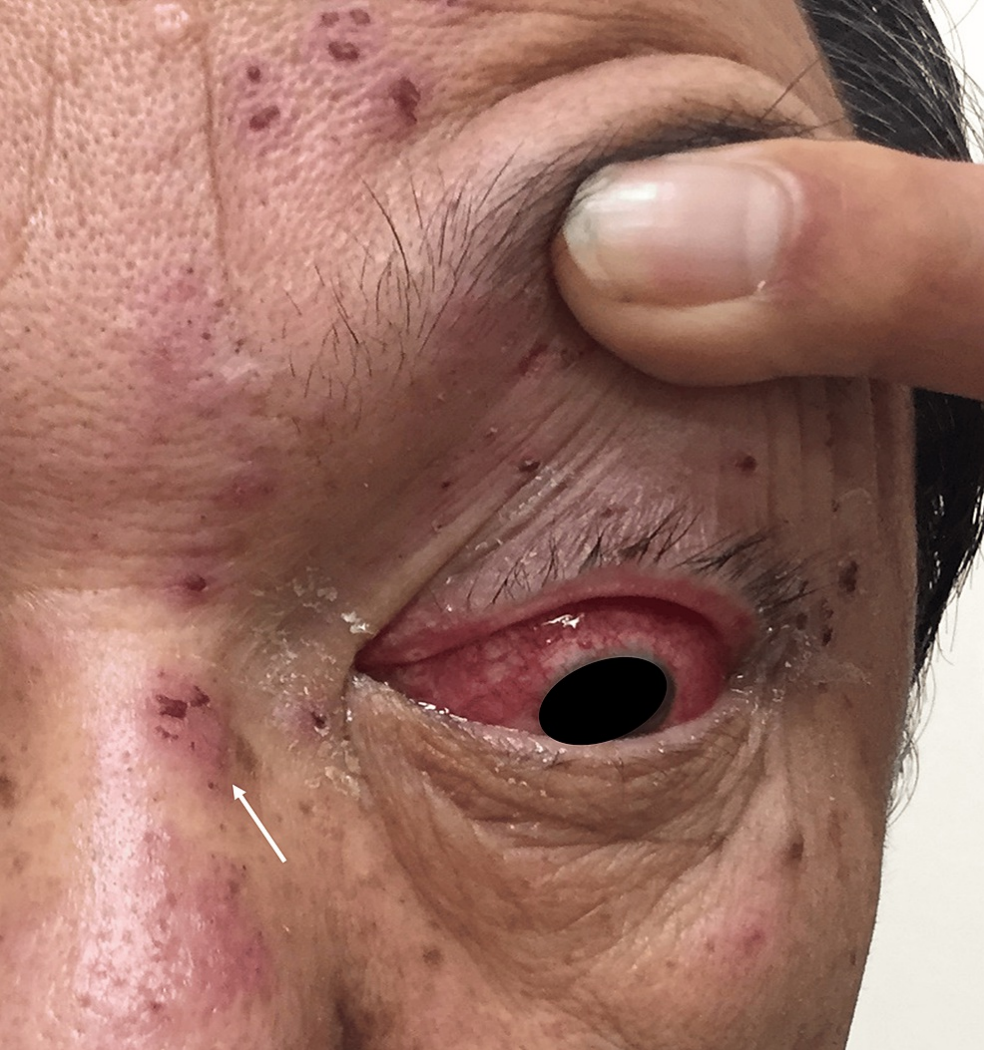
Ocular lesions and facial skin eruption thought to be Hutchinson's sign. The visage of the patient under scrutiny. A conspicuous discrepancy is evident when comparing the left and right sides of the face. The left side is distinguished by marked conjunctival injection and edematous eyelid. As shown in the arrow part, the presence of scabbed pigmented blisters is discerned along the left forehead, eyelid, and the lateral aspect of the nose. The patient has given written consent for this image to be published.

He presented alert and normothermic. The headache was markedly relieved. At the same time, there were obvious signs of hyperesthesia, hyperalgesia, and dysesthesia localized to the left frontal region, with discrete scabby herpetic eruptions (Figure 1). Importantly, meningeal signs were absent. We diagnosed him with HZO at this point.

On detailed ophthalmologic examination by an ophthalmologist, the patient's right eye was unremarkable, while his left eye showed a significant reduction in visual acuity to the point of only recognizing hand movements. In particular, the left eye was characterized by marked bulbar conjunctivitis, corneal opacity with associated edema, sluggish pupillary light reflex, and elevated intraocular pressure, measured at 36 mmHg. The range of motion of the left eye was significantly reduced, presumably due to intraorbital edema. The patient was diagnosed with secondary glaucoma secondary to HZO.

Laboratory studies upon admission to our hospital are shown in Table 1. Leukocytosis, possibly due to prednisolone administration, and an unremarkable C-reactive protein (CRP) level were seen. A lumbar puncture was performed, and CSF analysis revealed pleocytosis and a mildly elevated protein level. Of clinical significance, immunoglobulin G (IgG) and immunoglobulin M (IgM) levels directed against VZV in serum, and IgG levels directed against VZV in CSF were elevated. Based on these findings, the patient was diagnosed with VZV meningitis.

**Table 1:** Laboratory test results on admission. EIA: enzyme immunoassay; VZV: Varicella-zoster virus; IgM: immunoglobulin M; IgG: immunoglobulin G

Variables	Data	Reference range
Blood cells tests		
White blood cells	11.39	3.9-9.3 × 10^3^/µL
Neutrophils	83	40-74%
Lymphocytes	10	19-48%
Monocytes	4.0	3.4-9%
Eosinophils	1.0	0-7%
Basophils	1.0	0-2%
Myelocytes	1.0	0-5%
Diabetes-related tests		
HbA1c	7.1	4.6-6.2%
Random plasma glucose	190	70-120 mg/dL
Chemistry (serum)		
C-reactive protein	0.11	0.00-0.70 mg/dL
VZV IgM (EIA)	4.29	<0.8 EIA index
VZV IgG (EIA)	>128	<2.0 EIA index
CSF analysis		
Protein	70	15-50 mg/dL
Glucose	80	50-75 mg/dL
Leucocytes	91	0-2/µL
Mononuclear	91	0-2/µL
VZV IgM (EIA)	0.49	<0.8 EIA index
VZV IgG (EIA)	>12.8	<0.2 EIA index

A therapeutic regimen of intravenous acyclovir and methylprednisolone (1000 mg per day for three days) was initiated from the day of admission to our hospital, followed by a gradual taper of oral prednisolone, starting at 60 mg/day and decreasing weekly. After a three-day hospital stay, our ophthalmology team performed phacoemulsification and aspiration, culminating in the implantation of an intraocular lens in the left eye. With gratitude to the careful and intensive ophthalmologic intervention, the patient's left eye achieved a progressive recovery of visual acuity, eventually reaching a level of 20/20 vision. The visual field also returned to normal. The patient was discharged after a 15-day hospital stay marked by complete resolution of cephalalgia.

## Discussion

This patient was initially diagnosed with an inaugural manifestation of cluster headache, characterized by profoundly severe left-sided cephalalgia, ophthalmalgia, ipsilateral conjunctival injection, and eyelid edema. It is noteworthy to mention that a transient alleviation of symptoms, precipitated by the management of cluster headaches, potentially contributed to the diagnostic misstep. A course correction was not initiated until the emergence of cutaneous vesicles on the left upper face, an occurrence transpiring more than a week subsequent to the onset of the headache.

In retrospect, two salient observations challenged the veracity of the initial cluster headache diagnosis. Firstly, the age at onset of the majority of the patients with cluster headaches is thought to be under fourth decades [3]. Nevertheless, approximately 10% of cluster headache presentations unfold in individuals aged over 50 years [4]. Therefore, it is not possible to clearly classify whether a patient has cluster headache or not based on the age of onset. Secondly, the prototypical cluster headache symptomatology entails the spontaneous remission of both cephalalgia and ocular manifestations within a three-hour time frame (coded as 3.1 in the International Classification of Headache Disorders {ICHD}-3) [5]. In stark contrast, the patient under scrutiny endured persistent symptoms. Hence, an appreciation of this discrepancy becomes imperative in guiding a prompt and accurate diagnosis, culminating in appropriate therapeutic measures.

Manifestations typified by persistent cephalalgia and ipsilateral autonomic symptoms, confined unilaterally, necessitate differentiation from hemicrania continua (HC), as delineated in ICHD-3 under code 3.4 [5]. Given that many conditions can simulate HC symptoms, a comprehensive and meticulous diagnostic exclusion procedure is imperative [6]. It is pertinent to note that the diagnosis of HC hinges on a favorable response to indomethacin. However, it is incumbent upon clinicians to remain cognizant that an indomethacin response does not foreclose the possibility of secondary etiologies underpinning HC [7]. In this particular case, the patient presented with VZV meningitis devoid of meningeal signs. In VZV infection, which can produce nerve root inflammation and meningoencephalitis without skin rash, immunoglobulin titers (both IgG and IgM) and PCR for VZV in CSF are key to diagnosis [8]. In atypical clinical scenarios with severe pain, the prudent recourse to a cerebrospinal fluid examination becomes compelling.

## Conclusions

This report elucidates a case of painful trigeminal neuropathy attributed to acute Herpes zoster, initially erroneously diagnosed as cluster headache, primarily due to the delayed cutaneous eruption. It is imperative to differentiate HZO from cluster headache, especially in cases involving elderly patients or characterized by persistent cephalo-ophthalmalgia without typical fluctuation of symptoms. In the presence of clinical suspicion, CSF analysis should be prioritized for the evaluation of immunoglobulin titers (both IgG and IgM) and VZV PCR, with the overarching goal of expediting the correct diagnosis.
